# Census of solo LuxR genes in prokaryotic genomes

**DOI:** 10.3389/fcimb.2015.00020

**Published:** 2015-03-12

**Authors:** Sanjarbek Hudaiberdiev, Kumari S. Choudhary, Roberto Vera Alvarez, Zsolt Gelencsér, Balázs Ligeti, Doriano Lamba, Sándor Pongor

**Affiliations:** ^1^Protein Structure and Bioinformatics, International Center for Genetic Engineering and BiotechnologyTrieste, Italy; ^2^Faculty of Information Technology and Bionics, Pázmány Peter Catholic UniversityBudapest, Hungary; ^3^Istituto di Cristallografia, Consiglio Nazionale delle Ricerche, U.O.S di Trieste, Area Science ParkBasovizza, Trieste, Italy

**Keywords:** N-acyl homoserine lactone, quorum sensing, LuxR, solo, orphan

## Abstract

*luxR* genes encode transcriptional regulators that control acyl homoserine lactone-based quorum sensing (AHL QS) in Gram negative bacteria. On the bacterial chromosome, *luxR* genes are usually found next or near to a *luxI* gene encoding the AHL signal synthase. Recently, a number of *luxR* genes were described that have no *luxI* genes in their vicinity on the chromosome. These so-called solo *luxR* genes may either respond to internal AHL signals produced by a non-adjacent *luxI* in the chromosome, or can respond to exogenous signals. Here we present a survey of solo *luxR* genes found in complete and draft bacterial genomes in the NCBI databases using HMMs. We found that 2698 of the 3550 *luxR* genes found are solos, which is an unexpectedly high number even if some of the hits may be false positives. We also found that solo LuxR sequences form distinct clusters that are different from the clusters of LuxR sequences that are part of the known *luxR*-*luxI* topological arrangements. We also found a number of cases that we termed twin *luxR* topologies, in which two adjacent *luxR* genes were in tandem or divergent orientation. Many of the *luxR* solo clusters were devoid of the sequence motifs characteristic of AHL binding LuxR proteins so there is room to speculate that the solos may be involved in sensing hitherto unknown signals. It was noted that only some of the LuxR clades are rich in conserved cysteine residues. Molecular modeling suggests that some of the cysteines may be involved in disulfide formation, which makes us speculate that some LuxR proteins, including some of the solos may be involved in redox regulation.

## Introduction

Quorum sensing (QS) is a general intercellular signaling mechanism that allows bacterial populations to synchronize their behavior in a cell-density dependent manner (Fuqua et al., 1994; Miller and Bassler, 2001). Density dependent responses enable populations to solve problems that single bacterial cells cannot, such as the colonization of new habitats, infection of host organisms, etc. Originally studied in a few species only, a variety of QS mechanisms are now recognized throughout the entire bacterial world (Whitehead et al., 2001; Waters and Bassler, 2005; Case et al., 2008; Schaefer et al., 2008; Lindemann et al., 2011; Brachmann et al., 2013).

**Figure 1:**
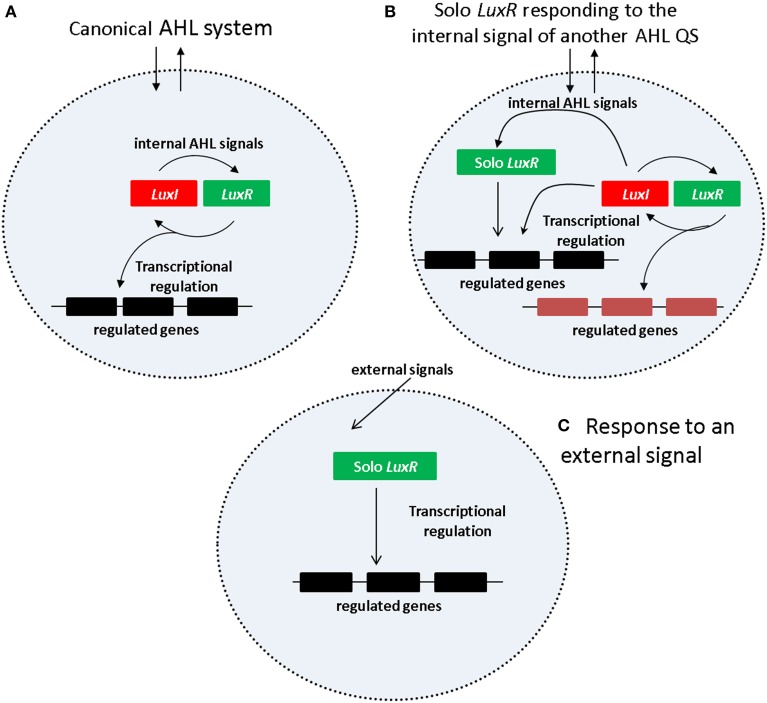
**Regulatory mechanism of AHL QS system. (A)** Canonical AHL system. **(B)** Solo LuxR responding to the signal of a nonadjacent LuxI synthase. **(C)** Solo LuxR responding to an external signal.

One of the simplest and the best studied among the QS mechanisms is *N-acyl Homoserine Lactone* (AHL) based signaling (briefly AHL QS) which is present in many Gram negative bacteria, including important human, animal and plant pathogens that occur in a wide variety of environments. In the AHL QS system (Figure 1A), AHL production is carried out by an AHL synthase that belongs to the LuxI protein family. The AHL molecules produced by *luxI* accumulate both inside and outside of cell membrane in equilibrium between the external and internal signal levels. The AHL molecules inside the cells bind to the signal receptor/regulator protein LuxR which will regulate transcription of both the *luxI* gene as well as other, downstream regulated genes. The *luxI* and *luxR* genes form a typical positive feedback loop usually referred to as an autoinduction circle, which is coupled to external signal concentration via the diffusible AHL molecules.

The regular arrangement of *luxI* and *luxR* genes was observed already in early studies. A review of Goryachev describes two canonical arrangements for *luxI* and *luxR* genes, a tandem arrangement (both genes on the same strand) and a convergent arrangement (with the two genes on opposite strands) (Goryachev, 2009). However, as more genome sequences became available, a number of further topological arrangements were found (Gelencsér et al., 2012a,b; Choudhary et al., 2013). Currently there are about 17 topologies known and it was also shown that the chromosomal neighborhood of AHL circuits contain a few recurrent elements, such as negative regulators of QS and genes involved in DNA mobilization.

Importantly it was also found that QS genes in a given local arrangement (topology) are apparent orthologs with respect to each other while they are paralogs with respect to *luxR* genes in different topological arrangements. For instance, the sequence of a LuxR protein within a tandem topology of *Burkholderia cepacia* is more similar to a LuxR protein of *P. aeruginosa* with the same topology than to another LuxR protein within its own genome which is part of a different type of chromosomal arrangement (such as RMI consisting of *luxR*, *rsaM* and *luxI*). In other words, AHL QS genes cluster according to topology which can be easily recognized in similarity cladograms.

An interesting subgroup of *luxR* genes are those which have no *luxI* gene in their chromosomal neighborhoods. The *qscR* gene of *P. aeruginosa LESB83* is a typical example, and C. Fuqua introduced the term “*orphan luxR*” for this gene in a seminal paper (Fuqua, 2006). As a large number of other genes were found subsequently in a variety of other regulatory and genomic contexts, the generic term solo was introduced for this larger group of genes (Subramoni and Venturi, 2009).

Current views suggest two kinds of regulatory scenarios for solo LuxR proteins (Figures 1B,C). In one of them (Figure 1B), the solo LuxR responds to the signal produced by an AHL QS circuit within the same cell. The *P.aeruginosa qscR* gene is an example of this scenario. In the other scenario (Figure 1C) the solo LuxR protein responds to an external signal which is not necessarily an AHL type molecule. Sequence conservation studies identified a number of conserved residues that are responsible for AHL binding (for a review see, Covaceuszach et al., 2013). Lamba and associates noticed that the AHL binding residues are conspicuously absent in a few solo LuxR proteins (Covaceuszach et al., 2013; Gonzalez and Venturi, 2013; Patel et al., 2014). On this basis, AHL-binding and non-AHL binding LuxR sequences can be tentatively distinguished. It was hypothesized that the identified proteins respond to external signals.

Identifying solo *luxR* genes in genomes is a delicate task, because the LuxR protein is structurally related to other, abundant protein families. Namely, LuxR is composed of two domains, the DNA-binding domain GerE (PFAM id: PF00196) and the autoinducer binding domain (PFAM id: PF03472). Both domains can be found in a variety of other proteins, for instance the GerE domain is part of 273 different types of protein architectures reviewed in the PFAM database (Finn et al., 2008). An ORF can be predicted as a genuine LuxR protein if it bears similarities to both domains, and in addition, the two domains should be in the right serial order, and the total length of the ORF must be in the range of known LuxR proteins (Gelencsér et al., 2012a,b; Choudhary et al., 2013). When looking for canonical QS circuits, false positives can be filtered out by requiring that *luxR* and *luxI* genes be within a certain distance on the chromosome (less than 3000 bp for simple topologies like RI, RMI, RLI and less than 3400 bp for RXMI topologies). When looking for solo *luxR* genes, we do not have such filtering criteria so there is a danger of accepting more false positives. In addition, a *luxR* gene may erroneously appear as a solo because one fails to detect the *luxI* gene in the vicinity, or because it is associated with a novel kind of signal synthase previously not recognized as a QS gene. Sequencing problems can easily cause such mistakes.

This article is concerned with the identification of solo *luxR* genes in the presently available bacterial genomes. We used rigorous criteria to screen complete and draft genomes, both at the proteome and at the DNA sequence level, and found that solo *luxR* genes are more frequent than previously thought. A large number of the solos are not likely to bind AHLs, so there is room for looking for new molecules binding to solo LuxR proteins. We also noted that a few groups of LuxR sequences contain a relatively large number of conserved cysteine residues and raised the hypothesis that they might be involved in sensing oxidative stress.

## Data and methods

For the purposes of the present survey we term a *luxR* gene a solo if it has no *luxI* gene in its vicinity (within 3000 bp up and downstream), and its chromosomal neighborhood is not obviously similar to any of the known AHL QS gene neighborhoods.

The genomic data used in this study were obtained from NCBI's publicly available repository of genomes. For mapping and identifying LuxR solo proteins, Hidden Markov Model (HMM) recognizers were used using Hidden Markov Model recognisers built using the HMMER program, HMMER 3.0 http://hmmer.janelia.org/, as described previously previously (Gelencsér et al., 2012a,b; Choudhary et al., 2013). We scanned 2771 complete and 6970 draft genomes, which in total contained around 25 million proteins.

## Results and discussion

### LuxR solos form separate clades

**Figure 2:**
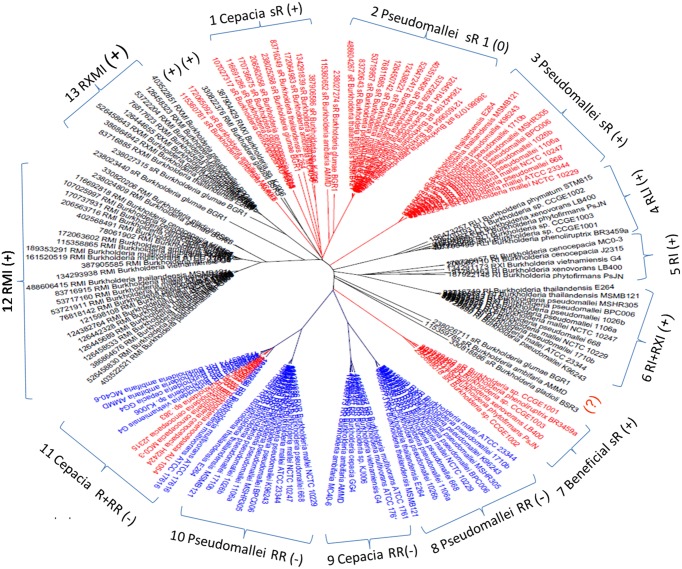
**Cladogram of *luxR* genes in the *Burkholderia* genus**. Red, solo *luxR*s; Blue, solo *luxR*s in twin topology; Black, *luxR* genes in known QS circuits. (+) and (−) indicates clades where the AHL binding motives are present and absent respectively. In the tags, R stands for *luxR*, I for *luxI*, M is *rsaM*, L is *rsaL* and X stands for any other gene between *luxR* and *luxI* (*X* genes are frequently hypothetical genes of unknown function). As a topological symbol of the clades, R stands for soloR, RR is twin topology, and RI is a canonical QS arrangement consisting of *luxR* and *luxI* genes. A detailed description of topological arrangements is given in (Gelencsér et al., 2012a,b; Choudhary et al., 2013).

Of 3550 *LuxR* genes (106 hypothetical) detected in total, 884 (21 hypothetical) were found to be member of AHL circuits and 2698 (85 hypothetical) were solo *luxR* genes, which makes 75% of all *LuxR* genes. The accession numbers of genes are given in Supplementary Material Table 1. LuxR protein sequences were previously shown to cluster according to the topological arrangement of the QS system genes. A sequence similarity clustering of all LuxR sequences showed that LuxR solos form separate clusters that are distinct from the LuxR sequences of complete QS systems. The entire cladogram is deposited in Supplementary Materials Data Sheet 1, a tree representing the *Burkholderia* genus is shown in Figure 2. It is apparent that solo LuxRs cluster separately and also that there are distinct types of LuxR solo sequences. This suggests that LuxR solos may be involved in distinct functions.

### Novel topological arrangements for LuxR solos

**Figure 3:**
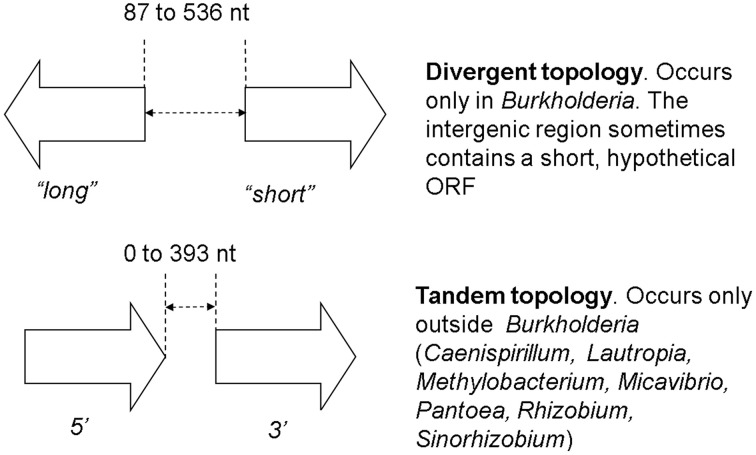
**Twin *luxR* arrangements and the suggested notation**. The genes occurring in these topologies are listed in Supplementary Materials Table 2. It is important to note that the **two** arrangements are taxonomically distinct, i.e., **one** taxon contains either **one** or the other.

While checking the local topologies in the clades of the *Burkholderia* tree (Figure 2) we discovered a novel topology type for solo *LuxR* genes, it was found that two solo *LuxR* genes are sometimes found adjacent to each other. We termed this new arrangement as the “twin *LuxR*” topology. We found two types of this arrangement, one of them is found in *Burkholderia*, the other one is in various other species (Figure 3).

These distributions of topologies are difficult to present for a large dataset, therefore for illustration purposes we show a cladogram for the *Burk holderia* genus (Figure 2). It is conspicuous that the solo *luxR* genes form separate clades and, same as for other topologies, so they are orthologous with respect to sequences within the clade and paralogous with respect to *luxR* genes present in different topologies, including those within the same genome. In other words we can conclude that at least some solo *luxR*s carry an independent function, so they evolve independently from other *luxR*s within the same genome.

### AHL binding motifs

In addition to local gene arrangements, we also found that LuxR sequences differ in terms of their characteristic sequence motifs. Previously, Venturi and associates observed a number of sequence motifs that characterize AHL-binding and non-AHL binding LuxR proteins, respectively (Covaceuszach et al., 2013; Gonzalez and Venturi, 2013; Patel et al., 2014). We tested these motifs and found that a sequence contains either an AHL-binding motif (one or more of 8 motifs), or a non-AHL binding motif (one or more of 3 motifs). We then added the respective labels to the sequences and found that if an AHL binding consensus motif is present in a sequence, it is conserved in the entire clade of the tree presented in Figure 2. On the contrary, if the AHL binding consensus motifs are absent, non-AHL binding motifs are inevitably present in the entire clade. On the one hand, this conveys confidence to the specificity of the motifs, on the other hand, the fact that only solo LuxR clades contain the non-AHL binding motifs supports the fact that some of the solo clades are involved in signaling other than AHL. There were a few sequences (outside the *Burkholderia* genus) that contained neither the AHL-binding, nor the non-AHL binding motif (Data Sheet 2). On the one hand, this fact points to the tentative nature of this kind of analysis. On the other hand, it can also point to novel signal types that are not similar to the ones originally included in the analysis of Venturi and associates (Covaceuszach et al., 2013; Gonzalez and Venturi, 2013; Patel et al., 2014).

### Cysteine residues—LuxR proteins as redox sensors?

We also observed that the LuxR proteins of the *Burkholderia* genus differ in terms of the number of cysteine residues (Table 1, column 4). Some of the clades shown in Figure 2 have 6 or 7 conserved cysteine residues while others have one or none. Characteristically, the numbers are conserved within the clades, so again we are tempted to believe that these differences may have a functional role. For instance, the one clade of solo LuxR proteins in *B. pseudomallei* has 7 cysteine residues (see multiple alignment in Supplementary Material Image 1), while another solo LuxR clade from *B. pseudomallei* has only 1. It is worth noting that the cysteine residues are mostly located within the autoinducer domain, not in the DNA binding domain.

**Table 1:** **Presence of AHL-binding and non-AHL-binding sequence motifs in the LuxR proteins in the Burkholderia genus**.

***LuxR* clade (in Figure 2)**	**AHL binding motif**	**Non-AHL binding motif**	**Cysteine content (min-max, average)**
RLI	+		4–4, 4
RI	+		2–3, 2.08
RI_RXI	+		3–7, 5.4
Beneficial	+		2–3, 2.17
Pseudomallei RR		+	4–6, 4.18
Cepacia-RR2		+	1–2, 1.71
Pseudomallei RR 2		+	4–6, 4.92
Cepacia_RR		+	1–2, 1.71
Cepacia_soloR		+	4–5, 4.2
RMI	+		2–6, 4.37
RXMI	+		0
Cepacia_soloR	+		2–5, 3.56
Pseudomallei_soloR 1			1–3, 1.23
Pseudomallei_soloR 2	+		7–14, 8

The asymmetric distribution of cysteines between clades and between protein domains makes us speculate about the potential functional role of the cysteine residues. One of the plausible ideas is disulfide-based redox regulation mediated by cysteines which is a well-known mechanism in bacterial transcription factors—for a review see (Ilbert et al., 2009). In theory, disulfide formation in a regulatory protein can reinforce active, folded conformations (up-regulation) or conversely, it can lock the protein into inactive, unfolded aggregates (down-regulation). A well-known example is the OxyR repressor, a LysR-type transcription factor that is responsible for the regulation of the antioxidant defense in a large variety of bacteria (Christman et al., 1989). Same as LuxR, OxyR consists of a helix-turn-helix type DNA-binding domain and another domain that mediates dimerization. Oxidative stress results in the formation of an intramonomeric disulfide bridge in the LysR domain, which activates OxyR ptoein by changing the DNA binding specificity. As a result, OxyR becomes rapidly activated and induces the transcription of its target genes (Storz and Tartaglia, 1992; Hausladen et al., 1996; Aslund and Beckwith, 1999). Interestingly, OxyR protein has 6 cysteines, out of which only 2 are involved in disulfide formation.

**Figure 4:**
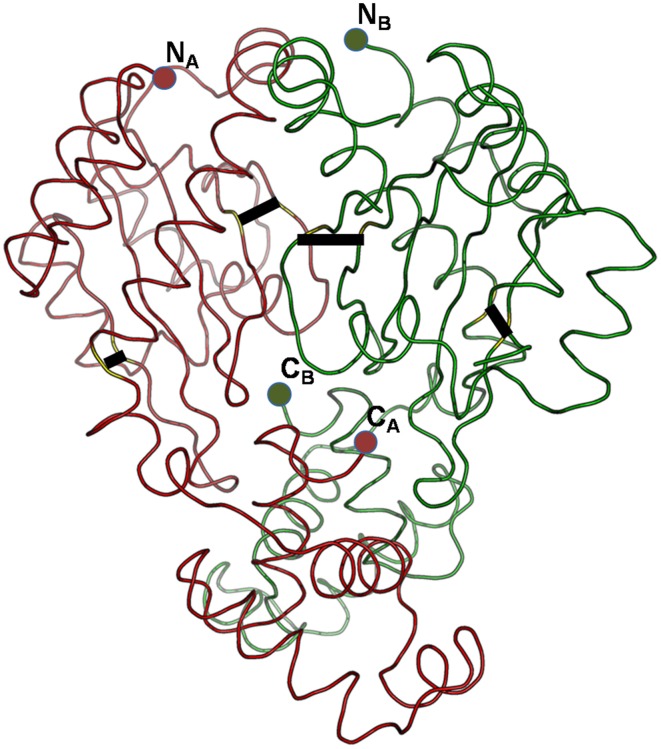
**Potential disulfide bridges in the *Burkholderia pseudomallei* solo LuxR proteins which form one clade on phylogenetic tree (Figure 2)**. Four potential disulfide bridges are mapped on the X-ray structure of the active conformation of the TraR dimer, which was crystallized along with the autoinducer (not shown) and target DNA (not shown) (PDB code: 1H0M). The four bridges are (from left to right): A48–A169, A82–A116, B82–B116, and B48–B169.

Can the cysteines conserved in LuxR proteins make disufile bonds that reinforce the active structure? This cannot be answered on a theoretical basis, but preliminary insights can be gained from the experimentally determined 3D structures of LuxR proteins. We used the crystal structure of the TraR protein of *Agrobacterium tumefaciens* (PDB code: 1H0M) as a template. In this structure the LuxR dimer is bound to AHL and to cognate DNA so this is an active conformation of a LuxR protein (Vannini et al., 2002). Consequently, if a conserved cystein pair in a LuxR homolog can be aligned with positions that are within the distance range of disulfide formation, the resulting disulfide bond can by definition reinforce the active conformation of the protein. We aligned all *Burkholderia* LuxR protein sequences to this template and determined whether or not the positions of conserved cysteine residues are within Cα-Cα distance range of disulfide formation (Table 2; the procedure is described in Supplementary Material Data Sheet 3). Interestingly, such disulfide bridge possibilities were found only among the solo LuxR proteins, and all of them were predicted within the autoinducer domain (). In the example shown in Figure 4, potential disulfide bridges found in *Burkholderia pseudomallei* solo LuxR proteins that form one clade on phylogenetic tree (Figure 2) were mapped on the X-ray structure of the active conformation of the TraR dimer. The highlighted disulfide bridges may thus stabilize the active conformation.

**Table 2:** **Potential disulfide bridges predicted for various clades of Burkholderia LuxR proteins**.

**Clade**	**Cys1^a^**	**Cys2**	**Cα-Cα distance [Angstrom]**
Cepacia solo	111	79	4.3
Pseudomallei long	107	91	4.1
Pseudomallei solo	116	78	4.4
	174	47	4.3

a *Positions given according to the numbering of the 1H0M PDB structure*.

*In silico* prediction of disulfide bridges *per se* cannot be regarded as a proof for LuxR proteins participating in redox responses. Nevertheless, the facts that, on the one hand, such predicted bridges were found only in solo LuxR proteins and only in their autoinducer domain and the analogy with the OxyR protein, on the other hand, makes us suggest that the role of some of the solo LuxRs in oxidative stress responses be further investigated in wet lab experiments.

## Conclusions

We present a large scale survey of *luxR* genes, trying to understand the mechanisms and phylogenic patterns of solo *luxR*s. We found that out of 3550 LuxR proteins found in the NCBI sequence repository, 2698 are solos, which is a surprisingly large number even if we suppose that some of these *luxR* solos may be associated with unknown or unidentified synthase genes. Transcriptional regulatory circuits can co-evolve independently from the target genes (Cases and De Lorenzo, 2005). Phylogenetic analysis of LuxRs (Figure 2) suggests that the evolution of solo LuxRs may be independent from the evolution of QS operons. The fact that taxonomically conserved solo LuxR proteins often contain non-AHL binding consensus motifs while QS-bound LuxRs tend to contain AHL-binding motifs, supports this hypothesis. Furthermore, we observed novel chromosomal arrangement pattern (topology) types, which we name *twin solo LuxRs* which is an addition to the arrangement types described previously (Gelencsér et al., 2012a,b; Choudhary et al., 2013). Last but not least, we hypothesize that some solo *luxR* genes might participate in redox regulation.

### Conflict of interest statement

The authors declare that the research was conducted in the absence of any commercial or financial relationships that could be construed as a potential conflict of interest.
