# Supplementary material for: Census of solo LuxR genes in prokaryotic genomes
Source: Front Cell Infect Microbiol. 2015 Mar 12;5:20. doi: 10.3389/fcimb.2015.00020 (PMC4357305; doi:10.3389/fcimb.2015.00020)
Supplement: Supplementary file 1 [file Table1.DOCX]

Table 1.Accession IDs of *luxR* genes which are parts of QS circuits and solo *luxR* genes.

| **Accesion numbers of *luxR*s from AHL circuits.** |
| --- |
| YP_972129, YP_002551488, YP_004088229, YP_001832058, YP_001241092, YP_001242900, NP_767702, YP_005605968, YP_001203095, YP_005454040, YP_002232873, YP_001777917, YP_106160, YP_001893790, YP_001076161, YP_335776, YP_001063209, YP_111575, YP_439002, YP_555670, YP_674864, YP_004752611, YP_001534186, YP_003538485, YP_003530769, YP_003740953, YP_005819077, YP_005803137, YP_002649216, YP_001908006, YP_007302795, YP_007307301, YP_007307631, YP_004144717, YP_004145052, NP_106261, NP_106660, NP_109411, YP_004611382, YP_004614647, YP_004615057, YP_001772212, YP_002423670, YP_002965846, YP_002966880, YP_003070967, YP_001641953, YP_002495629, YP_002497059, YP_001927660, YP_001758389, YP_001776815, YP_001783296, YP_002360441, YP_006590958, YP_317246, YP_004532623, YP_004534342, YP_005933615, YP_003519711, YP_005993449, YP_005196289, YP_004115278, YP_003930459, YP_006561608, YP_006563138, YP_006571841, YP_002128523, YP_004302430, YP_659944, YP_006481370, YP_002439139, YP_005974077, YP_005980556, YP_005982825, YP_001347033, NP_252167, YP_789670, YP_006324020, NP_522339, YP_003749681, YP_470410, YP_001979199, YP_002976727, YP_002282164, YP_768957, YP_002826207, YP_004012994, YP_781245, YP_530593, YP_567541, YP_569310, NP_945674, YP_004106680, YP_004106955, YP_004108424, YP_484040, YP_486928, YP_001989359, YP_001991323, YP_005417844, YP_428476, YP_006049737, YP_681951, YP_004691988, YP_165634, YP_167510, YP_528967, YP_005188893, YP_006399413, YP_001327236, NP_385944, YP_004549092, YP_005713746, YP_007190421, YP_006840442, YP_005720097, YP_003546444, YP_004835094, YP_004835756, YP_617565, YP_617627, YP_006374289, YP_007515595, YP_007516239, YP_008328695, YP_007920507, YP_007913656, YP_007916250, YP_007704445, YP_007706165, YP_007708313, YP_008134118, YP_008238175, YP_006060656, YP_008365652, YP_007575108, ZP_07025910, ZP_11426398, ZP_11178054, ZP_16040065, ZP_09473521, ZP_09420124, ZP_09438974, ZP_09426112, ZP_10081925, ZP_10085269, ZP_09646244, ZP_09651614, ZP_10584465, ZP_02891575, ZP_10034317, ZP_02885318, ZP_04812836, ZP_13109189, ZP_13115665, ZP_03456637, ZP_02452113, ZP_02460274, ZP_02494387, ZP_18330161, ZP_02466901, ZP_11134880, ZP_11135473, ZP_10019514, ZP_05779347, ZP_02165828, ZP_10320519, ZP_12691578, ZP_12693902, ZP_01004683, ZP_01011605, ZP_09295204, ZP_09295484, ZP_09296773, ZP_09085731, ZP_23960051, ZP_23962657, ZP_23963224, ZP_12697120, ZP_12697863, ZP_10355080, ZP_10358325, ZP_10182121, ZP_01044519, ZP_01127738, ZP_10744714, ZP_09192762, ZP_10362266, ZP_11130110, ZP_01155663, ZP_11147387, ZP_23288360, ZP_21148350, ZP_07379021, ZP_20937139, ZP_10221962, ZP_10557689, ZP_09929712, ZP_09510316, ZP_10551938, ZP_09051653, ZP_11921018, ZP_21934935, ZP_21936360, ZP_04935484, ZP_07794823, ZP_15612272, ZP_15618193, ZP_15624613, ZP_15631046, ZP_14646523, ZP_15636947, ZP_13150264, ZP_13155871, ZP_18358620, ZP_06877480, ZP_01364783, ZP_01366928, ZP_14281273, ZP_15964692, ZP_21956730, ZP_18875661, ZP_10175258, ZP_10708649, ZP_10148853, ZP_01113402, ZP_10835124, ZP_10734254, ZP_11035428, ZP_11035750, ZP_03511447, ZP_18319688, ZP_18342196, ZP_18306179, ZP_18295286, ZP_16036154, ZP_05123460, ZP_05123091, ZP_05076090, ZP_05076097, ZP_01740882, ZP_05077838, ZP_05078496, ZP_05845130, ZP_01901710, ZP_01751729, ZP_05098927, ZP_01057534, ZP_01755720, ZP_01038174, ZP_05090397, ZP_08862066, ZP_08862837, ZP_01746123, ZP_06637774, ZP_05785479, ZP_05787303, ZP_05742871, ZP_12977252, ZP_10544478, ZP_18899998, ZP_18901624, ZP_09140922, ZP_10871198, ZP_01303368, ZP_00964786, ZP_05344165, ZP_11123509, YP_002541325, YP_002923741, NP_903760, YP_003366469, YP_002955225, YP_003885142, YP_003331714, YP_003002472, YP_002934275, YP_003296639, YP_005699929, YP_003941575, YP_003740504, YP_001906896, YP_002537872, YP_001231850, YP_005934164, YP_003520251, YP_005992881, YP_005195655, YP_003729882, YP_048234, YP_003019697, YP_006644751, YP_003261727, YP_234708, YP_273861, NP_793635, YP_005200648, YP_531902, YP_001476304, YP_453965, YP_002947664, YP_004577680, YP_005049773, YP_001005891, YP_004298720, YP_006003987, YP_005519720, YP_005522421, YP_001604810, YP_001606210, YP_650193, YP_651866, YP_005622789, YP_005623571, NP_993605, NP_994736, YP_002346032, YP_002347421, YP_005503920, YP_005505358, YP_005507791, YP_005509307, NP_669049, NP_670674, YP_647982, YP_649110, YP_001161917, YP_001163230, YP_003566925, YP_003568279, YP_001399708, YP_001400524, YP_071012, YP_071752, YP_001873010, YP_001873807, YP_001719545, YP_001720401, YP_007629974, YP_006285524, YP_008136314, YP_008156902, YP_008156948, ZP_09015053, ZP_08914854, ZP_07395071, ZP_11316110, ZP_06715736, ZP_21919334, ZP_10412528, ZP_07949954, ZP_10810680, ZP_09379112, ZP_09511415, ZP_09828348, ZP_03828135, ZP_15540199, ZP_18878111, ZP_10177171, ZP_10177208, ZP_10708399, ZP_16735510, ZP_16659589, ZP_06480662, ZP_06457463, ZP_17804161, ZP_17808771, ZP_11562922, ZP_11571343, ZP_16669549, ZP_16679858, ZP_16665628, ZP_16741531, ZP_05641622, ZP_07254407, ZP_07231401, ZP_07257647, ZP_10538611, ZP_11268340, ZP_09968803, ZP_09972201, ZP_23062130, ZP_06192455, ZP_06637789, ZP_16217530, ZP_05878949, ZP_09363604, ZP_01865484, ZP_20504167, ZP_12867815, ZP_14761677, ZP_02238708, ZP_02240585, ZP_02229087, ZP_02229724, ZP_02316949, ZP_02318768, ZP_02223301, ZP_04462686, ZP_04513696, ZP_02227832, ZP_02228021, ZP_02311312, ZP_02314056, ZP_04458148, ZP_04461067, ZP_01887669, ZP_01889042, ZP_02333326, ZP_02334325, ZP_16199230, ZP_16200742, ZP_06204666, ZP_04517664, ZP_04518921, ZP_04456830, ZP_04510361, ZP_15043677, ZP_15045302, ZP_15048428, ZP_15050059, ZP_15058200, ZP_15059785, ZP_15062739, ZP_15064358, ZP_15067874, ZP_15069483, ZP_15072707, ZP_15074303, ZP_15077559, ZP_15079147, ZP_15082203, ZP_15083758, ZP_15302382, ZP_15303953, ZP_15307036, ZP_15308650, ZP_15312110, ZP_15313738, ZP_15088616, ZP_15323116, ZP_15093640, ZP_15096977, ZP_15098535, ZP_15101802, ZP_15103402, ZP_15111283, ZP_15112854, ZP_15115869, ZP_15117468, ZP_15122055, ZP_15125117, ZP_15126732, ZP_15130173, ZP_15131754, ZP_15134865, ZP_15136497, ZP_15139937, ZP_15141566, ZP_15144892, ZP_15146492, ZP_15149657, ZP_15151269, ZP_15154156, ZP_15155714, ZP_15158984, ZP_15160566, ZP_15164015, ZP_15165633, ZP_15170357, ZP_15173318, ZP_15174935, ZP_15180014, ZP_15182985, ZP_15187881, ZP_15189442, ZP_15192659, ZP_15194268, ZP_15197391, ZP_15202209, ZP_15203815, ZP_15207220, ZP_15208846, ZP_15211790, ZP_15213380, ZP_15216723, ZP_15218304, ZP_15221413, ZP_15223089, ZP_15226370, ZP_15227983, ZP_15230934, ZP_15235719, ZP_15237251, ZP_15240450, ZP_15241936, ZP_15244932, ZP_15246456, ZP_15249777, ZP_15251365, ZP_15256271, ZP_15259321, ZP_15260921, ZP_15264266, ZP_15265879, ZP_15268868, ZP_15270419, ZP_15273682, ZP_15275288, ZP_15278440, ZP_15280067, ZP_15283548, ZP_15285075, ZP_15288230, ZP_15289848, ZP_15292784, ZP_15294407, ZP_15298003, ZP_15299552, YP_855090, YP_001143472, YP_004391246, YP_002265247, YP_001220570, YP_003744152, NP_521406, YP_006028009, YP_003750859, YP_003558208, YP_001761363, YP_206883, YP_002158591, YP_008041702, YP_007510517, YP_007513947, YP_007634343, YP_005995203, ZP_11387263, ZP_08518815, ZP_17186019, ZP_15943031, ZP_12961851, ZP_17197570, ZP_17187751, ZP_17191832, ZP_11085677, ZP_08869914, ZP_10465110, ZP_17691913, ZP_09472223, ZP_09420978, ZP_10079730, ZP_10702165, ZP_10682927, ZP_16321000, ZP_00944450, ZP_01036535, ZP_17662991, YP_473056, YP_001985289, YP_008369033, ZP_08645811, ZP_10271451, ZP_03524578, ZP_18311267, ZP_18320087, ZP_18341868, ZP_18307671, YP_002426403, YP_002220093, YP_005512716, YP_002317567, YP_002327279, YP_001844797, YP_001083200, YP_001715477, YP_006291536, YP_005524096, YP_005797026, YP_006846671, YP_004997306, YP_003734010, YP_371810, YP_776003, YP_001811253, YP_623508, YP_838351, YP_002234479, YP_001779191, YP_006617835, YP_004349068, YP_004362597, YP_002909041, YP_006335923, YP_105961, YP_001024423, YP_001078154, YP_989940, YP_001583946, YP_001948918, YP_006277624, YP_001075258, YP_337635, YP_001062292, YP_006658090, YP_110896, YP_439706, YP_001117674, YP_003262848, YP_003624229, YP_008207613, YP_008215307, YP_008211481, YP_008327958, YP_007922136, ZP_08438707, ZP_08434826, ZP_08443403, ZP_22619373, ZP_23047950, ZP_22015943, ZP_21459915, ZP_17791652, ZP_22650196, ZP_22816987, ZP_23191861, ZP_22061557, ZP_22455497, ZP_22666442, ZP_22529880, ZP_23206750, ZP_22525005, ZP_23212475, ZP_23140505, ZP_23035251, ZP_23148523, ZP_22083395, ZP_22313719, ZP_22543971, ZP_22515384, ZP_21982020, ZP_11601145, ZP_17799037, ZP_17802606, ZP_23039549, ZP_22588330, ZP_22211474, ZP_22095190, ZP_22304422, ZP_04662393, ZP_22519847, ZP_22408653, ZP_12515069, ZP_12520789, ZP_12521977, ZP_12528209, ZP_23090815, ZP_22804750, ZP_23232222, ZP_22207106, ZP_22561829, ZP_22369783, ZP_22394278, ZP_22866676, ZP_22833072, ZP_22387849, ZP_23245532, ZP_15659087, ZP_15980485, ZP_05829841, ZP_16237840, ZP_12225879, ZP_16129692, ZP_15905989, ZP_16128714, ZP_16082666, ZP_16087015, ZP_16140345, ZP_22873714, ZP_16074971, ZP_12228009, ZP_12199780, ZP_16234556, ZP_16229908, ZP_16097191, ZP_21453852, ZP_12205599, ZP_16098095, ZP_16091092, ZP_12196101, ZP_16245923, ZP_21447569, ZP_16112903, ZP_16063389, ZP_16106706, ZP_16111885, ZP_15657063, ZP_16103541, ZP_16118894, ZP_12210731, ZP_12218382, ZP_16072168, ZP_12221625, ZP_21449625, ZP_23006134, ZP_22463901, ZP_22167336, ZP_23175078, ZP_23009256, ZP_18914774, ZP_16133838, ZP_16241385, ZP_21444416, ZP_16141225, ZP_16144974, ZP_06059129, ZP_06693484, ZP_02888313, ZP_02891331, ZP_02905627, ZP_02911189, ZP_21159968, ZP_16299446, ZP_21172668, ZP_04947566, ZP_04973300, ZP_04880817, ZP_04907171, ZP_00442030, ZP_04911129, ZP_02265991, ZP_15917554, ZP_03582735, ZP_15923845, ZP_02365558, ZP_02358494, ZP_04812802, ZP_02501582, ZP_02414978, ZP_04889933, ZP_04953922, ZP_01765698, ZP_03449846, ZP_02485226, ZP_02451058, ZP_02459223, ZP_02474734, ZP_02509481, ZP_02406468, ZP_23812404, ZP_04520133, ZP_02493373, ZP_03793118, ZP_04899529, ZP_02384911, ZP_05591107, ZP_18328617, ZP_02466172, ZP_02371020, ZP_02376825, ZP_08780482, ZP_10994633, ZP_10189944, YP_004487116, YP_006277890, YP_001075604, YP_335301, YP_001062648, YP_006658420, YP_111189, YP_439427, YP_008328242, ZP_13101816, ZP_04812005, ZP_02502038, ZP_13109046, ZP_13115255, ZP_04953778, ZP_01770091, ZP_12970116, ZP_13120624, ZP_04967975, ZP_03450700, ZP_02485632, ZP_02475139, ZP_02509873, ZP_23814337, ZP_04522785, ZP_02493793, ZP_03795798, ZP_04899932, ZP_02384629, ZP_05590813, ZP_18330499, ZP_02466494, ZP_02370744, YP_001603072, YP_004551487, ZP_01896607, YP_001025820, YP_001077903, YP_006278304, YP_006658956, YP_008404994, ZP_04907393, ZP_00439362, ZP_04911346, ZP_02268175, ZP_02366208, ZP_02359139, ZP_13102245, ZP_02502639, ZP_02416029, ZP_04955166, ZP_12970525, ZP_13121017, ZP_02486263, ZP_02475767, ZP_02510483, ZP_02407518, ZP_23813306, ZP_03791573, ZP_02384183, ZP_05590371, ZP_02370271, ZP_11339768, ZP_01012895, ZP_18308876, ZP_18309819, ZP_08872887, YP_002496262, YP_007594669, YP_007467288, ZP_08745845, ZP_08752466, ZP_08749211, YP_001114942, ZP_04893413, ZP_01880907, ZP_04896779, YP_004688781, YP_004230807, YP_003608086, YP_003910271, YP_006793363, YP_001860599, YP_001888024, YP_554691, YP_003847232, YP_002441567, YP_005976284, YP_001349253, NP_250121, YP_791822, YP_007710453, ZP_14754745, ZP_10032579, ZP_02884277, ZP_03267650, ZP_10252321, ZP_18893180, ZP_09054016, ZP_11912347, ZP_11918768, ZP_04933102, ZP_07792658, ZP_15614371, ZP_15620740, ZP_15627053, ZP_04927852, ZP_15633176, ZP_14644725, ZP_15639092, ZP_13147355, ZP_13156465, ZP_18356395, ZP_22977885, ZP_06879678, ZP_14279254, ZP_15962750, ZP_21954534, ZP_10990415, YP_006334767 |
| **Accesion numbers of solo *luxR*s** |
| YP_003978623, YP_004783944, YP_004234977, YP_045866, YP_855658, YP_858670, YP_001143989, YP_004394647, YP_004277957, YP_004279508, YP_004279964, YP_004280354, YP_002541315, YP_002543565, YP_002545189, YP_002546300, YP_002551281, YP_002551307, YP_002540056, YP_002540155, YP_002542675, YP_002547516, YP_002548652, YP_002550142, YP_002550357, YP_002551190, YP_002265254, YP_195352, YP_003450678, YP_001833603, YP_001240084, NP_767883, NP_768520, YP_005612783, YP_005113710, YP_005150476, YP_220957, YP_222926, YP_001932080, YP_001934208, YP_001592065, YP_001594092, YP_005115475, YP_005153227, YP_002731954, YP_002733953, YP_413678, YP_418357, NP_540675, NP_542094, YP_005596287, YP_005598378, YP_005599647, YP_005601741, YP_005602996, YP_005660504, YP_003104919, YP_003106160, YP_001257186, YP_001258220, YP_004755275, YP_004757345, YP_005613833, YP_005615047, NP_697228, NP_699323, YP_001621970, YP_001626863, YP_005108139, YP_005153895, YP_371614, YP_776923, YP_777789, YP_777918, YP_001815695, YP_001815804, YP_004231614, YP_003606911, YP_003909532, YP_623375, YP_624828, YP_836819, YP_838485, YP_002232821, YP_002234663, YP_001777935, YP_001779043, YP_004351103, YP_002907673, YP_002909500, YP_002910942, YP_002911505, YP_002911546, YP_006336923, YP_102421, YP_106048, YP_001025964, YP_001028861, YP_001078053, YP_001078722, YP_001081227, YP_993677, YP_006794162, YP_001889745, YP_006274028, YP_006277017, YP_001066980, YP_001074487, YP_334182, YP_337020, YP_001059696, YP_001061548, YP_006653540, YP_006657283, YP_108943, YP_110332, YP_439875, YP_440275, YP_442349, YP_001115608, YP_553061, YP_002924396, YP_003796288, YP_001682990, YP_001683742, YP_003594859, YP_001981723, YP_673968, YP_001452611, YP_003365526, YP_004750813, YP_003278638, YP_001437405, YP_006342612, YP_003210982, YP_002007906, YP_004111977, YP_003882209, YP_003334459, YP_002985768, YP_001532893, YP_001177223, YP_004594785, YP_004829147, YP_003611882, YP_006478282, YP_004952709, YP_006579275, YP_003941366, YP_007339897, YP_006319158, YP_006096385, YP_002403190, YP_006106236, YP_852971, YP_001724700, YP_003035962, YP_002926921, NP_754222, YP_006154256, YP_006149337, YP_006091678, YP_006129257, YP_001463220, YP_002398121, YP_006115591, YP_001458706, YP_002387413, YP_002407149, YP_006101214, YP_001730884, NP_416426, YP_490173, YP_006164953, YP_005277539, YP_002556791, YP_006139108, YP_003222098, YP_006769651, YP_006783776, YP_006778705, YP_003234910, YP_002329552, YP_002271030, NP_288377, NP_310681, YP_003078403, YP_003229787, YP_003499911, YP_006159414, YP_006144268, YP_006120269, YP_006168186, YP_002391687, YP_002293422, YP_003349758, YP_001743327, YP_006110423, YP_002412930, YP_006134139, YP_541121, YP_006124785, YP_006173495, YP_006313319, YP_002382338, YP_004583344, YP_005377366, YP_006720480, YP_004866957, YP_004869123, YP_003267496, YP_003059176, YP_509095, YP_510243, YP_511135, YP_006499419, YP_006499611, YP_005020778, YP_005021014, YP_006635622, YP_002237709, YP_005227735, YP_005955462, YP_001336067, YP_002920209, YP_003438628, YP_003456134, YP_005469418, YP_007304468, YP_007307388, YP_007307461, YP_004141743, YP_004144803, YP_004144877, NP_106527, YP_004611293, YP_004614734, YP_004614811, YP_001939232, YP_001022984, YP_002496314, YP_002363616, YP_571814, YP_571851, YP_571965, YP_413098, YP_001165935, YP_001166164, YP_004538696, YP_001368758, YP_001369224, YP_001372753, YP_005946052, YP_005952341, YP_002290602, YP_004634329, YP_004638514, YP_004117160, YP_004118484, YP_917487, YP_001412948, YP_049663, YP_003017024, YP_006646115, YP_003259225, YP_004900611, YP_006562795, YP_006589903, YP_006564721, YP_006572987, YP_002130862, NP_927679, NP_931726, YP_552230, YP_004302132, YP_004302243, YP_006483213, YP_006483971, YP_002441012, YP_002441767, YP_005975729, YP_005976479, YP_005980252, YP_005981053, YP_001348746, NP_249827, NP_250589, YP_791341, YP_792130, YP_004351870, YP_004353552, YP_004356318, YP_608439, YP_610214, YP_006322860, YP_006323189, YP_006325836, YP_006325991, YP_006326038, YP_005206217, YP_005208986, YP_005210452, YP_347979, YP_350617, YP_260729, YP_262370, YP_002871035, YP_002871271, YP_002874841, YP_006385638, YP_005932122, YP_006531470, YP_001269813, YP_001670863, YP_007231335, NP_746756, YP_004703888, YP_007030036, YP_007031702, YP_001747665, YP_234943, YP_237284, YP_274051, YP_276356, NP_791871, NP_794292, YP_005080062, YP_005083391, YP_005220344, YP_005418966, YP_004215311, YP_001898047, YP_468171, YP_468395, YP_468692, YP_470573, YP_471472, YP_471858, YP_001976871, YP_001977110, YP_001977404, YP_001979365, YP_001980395, YP_001984868, YP_002974168, YP_002974391, YP_002976882, YP_002977948, YP_002978702, YP_002978861, YP_002279812, YP_002280028, YP_002280317, YP_002281222, YP_002282314, YP_002283328, YP_002284132, YP_765467, YP_766287, YP_766531, YP_769108, YP_770203, YP_770451, YP_771022, NP_444035, NP_443817, YP_002827770, YP_007332782, YP_007334523, YP_007335530, YP_007335846, YP_007336143, YP_007336346, YP_007336629, YP_003577255, YP_003577975, YP_351789, YP_355176, YP_001170341, YP_001042275, YP_001045267, YP_002520829, YP_002524401, YP_780214, YP_532083, YP_005416271, YP_427022, YP_427266, YP_006048252, YP_006048506, YP_684107, YP_004689462, YP_005437302, YP_167002, YP_167209, YP_611338, YP_613207, YP_004730634, YP_002146073, YP_216941, YP_002215136, YP_002243187, YP_002226156, YP_005213000, YP_006088179, YP_002046001, YP_002041215, YP_002141698, YP_150210, YP_001587454, YP_002637347, YP_002114983, NP_456513, YP_005237917, YP_005397312, NP_460903, YP_005181773, YP_005242857, YP_005247667, YP_005252456, YP_005216571, NP_804754, YP_006885426, YP_004498549, YP_004500218, YP_004500413, YP_006022964, YP_006024632, YP_006024827, YP_007345183, YP_004503501, YP_004505170, YP_004505366, YP_001478064, YP_001879705, YP_407563, YP_005727635, NP_837531, NP_707803, YP_689409, YP_005455844, YP_310157, YP_003524886, YP_005190623, YP_005193122, YP_006401062, YP_001314089, YP_001314420, YP_001328266, YP_001328801, NP_386820, NP_386921, NP_387385, YP_004550090, YP_004550191, YP_004550595, YP_005714685, YP_005714786, YP_005715189, YP_007191344, YP_007191444, YP_006813576, YP_006841442, YP_006841544, YP_006841949, YP_005721383, YP_005721487, YP_005721901, YP_005724882, YP_455420, YP_004553085, YP_003543845, YP_004836455, YP_001260394, YP_001260684, YP_001262495, YP_001264837, YP_006184493, YP_004792178, YP_001971663, YP_002027963, YP_002891515, YP_004157239, YP_004157258, YP_005335334, YP_005632521, YP_002812464, YP_002876204, YP_004939546, NP_233273, YP_002821981, YP_001215191, YP_001409333, NP_643297, YP_004852473, YP_242384, NP_638166, YP_005638172, YP_364866, YP_199907, YP_450196, YP_005627771, YP_001915061, YP_001005359, YP_008042324, YP_008045532, YP_007551548, YP_008327337, YP_008343262, YP_007917786, YP_007920870, YP_007440171, YP_007844665, YP_008106911, YP_008110012, YP_007557213, YP_008424858, YP_007641730, YP_007698699, YP_007699525, YP_008404153, YP_008408453, YP_006282712, YP_007709968, YP_007710751, YP_008131685, YP_008132421, YP_008000939, YP_008002517, YP_007396483, YP_007398991, YP_008097291, YP_008111747, YP_008101186, YP_007872784, YP_008363422, YP_008363647, YP_008363934, YP_008365808, YP_008366737, YP_008366979, YP_008368048, YP_008368586, YP_008322694, YP_008258288, YP_008308270, YP_008261215, YP_008247859, YP_008253149, YP_007470103, YP_008360964, YP_008381140, YP_007902327, YP_008254846, YP_007925271, YP_008228074, YP_008229659, YP_007404323, YP_007405603, YP_007407047, YP_007408490, YP_008136268, YP_008137848, YP_008157298, YP_008158654, YP_007575990, YP_007576091, YP_007576563, YP_008156438, YP_007637426, YP_007651239, ZP_08646815, ZP_10390020, ZP_10393281, ZP_07229233, ZP_07238678, ZP_07242212, ZP_23252982, ZP_21995422, ZP_22708626, ZP_22898520, ZP_21429575, ZP_10905734, ZP_10939163, ZP_09142040, ZP_21437122, ZP_11386727, ZP_11387240, ZP_11387741, ZP_08518263, ZP_08521822, ZP_17184377, ZP_17185542, ZP_15943550, ZP_15946177, ZP_12963892, ZP_17198195, ZP_17189583, ZP_17191247, ZP_17194413, ZP_11082274, ZP_11083222, ZP_11083942, ZP_11431376, ZP_11173581, ZP_11173611, ZP_11173926, ZP_11175218, ZP_11175375, ZP_11176147, ZP_08527020, ZP_08528880, ZP_08529992, ZP_08530499, ZP_23999609, ZP_24000311, ZP_24001594, ZP_24002806, ZP_24002828, ZP_24002845, ZP_24003589, ZP_12908339, ZP_12908901, ZP_12909732, ZP_12911814, ZP_12503321, ZP_12504113, ZP_12504343, ZP_12507809, ZP_12508003, ZP_12508067, ZP_12980589, ZP_12982504, ZP_12983755, ZP_12983781, ZP_12984082, ZP_07376030, ZP_11147991, ZP_21950489, ZP_01228887, ZP_16044908, ZP_09472701, ZP_09646099, ZP_09014992, ZP_06096103, ZP_06098417, ZP_04593655, ZP_17166027, ZP_17167585, ZP_17169153, ZP_17170712, ZP_17173155, ZP_17173838, ZP_17176255, ZP_17177629, ZP_17154374, ZP_17155755, ZP_17156628, ZP_17158188, ZP_17161425, ZP_17161725, ZP_17163299, ZP_17164738, ZP_05873335, ZP_05875147, ZP_05929603, ZP_05929908, ZP_05871618, ZP_05871919, ZP_06931299, ZP_06933683, ZP_05868394, ZP_05869406, ZP_05894482, ZP_05896681, ZP_05820133, ZP_05822202, ZP_07477047, ZP_07478868, ZP_07473798, ZP_07475179, ZP_05934269, ZP_05935697, ZP_03784765, ZP_03786630, ZP_05931042, ZP_05932470, ZP_06109224, ZP_06109935, ZP_05958825, ZP_05960160, ZP_06003013, ZP_05833747, ZP_05834759, ZP_06102037, ZP_06104948, ZP_05465427, ZP_06111771, ZP_06105978, ZP_06106691, ZP_05962805, ZP_05963501, ZP_07470241, ZP_07472955, ZP_06792262, ZP_06793815, ZP_05956043, ZP_05958188, ZP_05952967, ZP_05953795, ZP_06099563, ZP_06100474, ZP_05999789, ZP_05837644, ZP_05839014, ZP_05993590, ZP_05995224, ZP_02888481, ZP_02905153, ZP_02909525, ZP_14754069, ZP_21163207, ZP_21164999, ZP_16295840, ZP_21168070, ZP_21173120, ZP_04942658, ZP_04943512, ZP_10030988, ZP_10037452, ZP_02883785, ZP_03267716, ZP_09749682, ZP_04974064, ZP_04975254, ZP_04975384, ZP_04883242, ZP_04882899, ZP_04884441, ZP_04905688, ZP_04907266, ZP_00442115, ZP_00439585, ZP_04911220, ZP_04911987, ZP_02269385, ZP_02269610, ZP_02363469, ZP_02364030, ZP_02365046, ZP_02356337, ZP_02356927, ZP_02357886, ZP_02359024, ZP_13100941, ZP_13102887, ZP_04816106, ZP_04813012, ZP_02498852, ZP_02500748, ZP_02501250, ZP_02501253, ZP_13105906, ZP_13108200, ZP_13111812, ZP_13114386, ZP_02412205, ZP_02414126, ZP_02414631, ZP_02414635, ZP_02415394, ZP_04890894, ZP_04891720, ZP_04952059, ZP_04953780, ZP_01764525, ZP_01765989, ZP_01768685, ZP_01770887, ZP_12966445, ZP_12969246, ZP_13117678, ZP_13123075, ZP_04964417, ZP_04966879, ZP_04968860, ZP_03452593, ZP_03454727, ZP_02482528, ZP_02484382, ZP_02484901, ZP_02484905, ZP_02448314, ZP_02450208, ZP_02450718, ZP_02450723, ZP_02451465, ZP_02456509, ZP_02458381, ZP_02458896, ZP_02459643, ZP_02472054, ZP_02473938, ZP_02474408, ZP_02506853, ZP_02508690, ZP_02509164, ZP_02403673, ZP_02406115, ZP_02406889, ZP_23810934, ZP_23812127, ZP_04520705, ZP_02490715, ZP_02492564, ZP_02493063, ZP_03790147, ZP_03795550, ZP_04895885, ZP_04897839, ZP_04900889, ZP_04901665, ZP_04902374, ZP_02385155, ZP_02385619, ZP_02387967, ZP_05586811, ZP_05589165, ZP_05591292, ZP_18327042, ZP_18330227, ZP_02464119, ZP_02465600, ZP_02465921, ZP_02371258, ZP_02371348, ZP_02371764, ZP_02374125, ZP_02376379, ZP_02381677, ZP_02382407, ZP_02382558, ZP_18917326, ZP_18918694, ZP_10751858, ZP_11132655, ZP_11132704, ZP_11133146, ZP_10136060, ZP_10018094, ZP_05780138, ZP_05784535, ZP_04562443, ZP_10410027, ZP_09336017, ZP_16278981, ZP_22994775, ZP_06352425, ZP_06860759, ZP_06863193, ZP_13096224, ZP_07043189, ZP_12439650, ZP_16183603, ZP_16187666, ZP_07334483, ZP_10410501, ZP_05968172, ZP_14474430, ZP_08498664, ZP_09038272, ZP_10488709, ZP_10490764, ZP_10807783, ZP_01039636, ZP_01862490, ZP_01862615, ZP_01865189, ZP_10980476, ZP_04536353, ZP_10058635, ZP_02903853, ZP_22850563, ZP_23028825, ZP_22992360, ZP_11442575, ZP_18803926, ZP_18809619, ZP_18689998, ZP_21244567, ZP_16254157, ZP_18538901, ZP_18544439, ZP_03071636, ZP_11989866, ZP_11966852, ZP_07788045, ZP_07781335, ZP_12046747, ZP_12059408, ZP_12242767, ZP_12071027, ZP_18679691, ZP_12247746, ZP_12064055, ZP_12006996, ZP_12053073, ZP_11487167, ZP_18501158, ZP_21360769, ZP_12012924, ZP_12040784, ZP_11980116, ZP_12033104, ZP_18506880, ZP_02999201, ZP_14440418, ZP_14448213, ZP_18659257, ZP_14449791, ZP_18642377, ZP_21339829, ZP_14458673, ZP_16248345, ZP_18517107, ZP_18521723, ZP_18527426, ZP_18532960, ZP_04003462, ZP_18550095, ZP_19025354, ZP_19019665, ZP_19030808, ZP_19042153, ZP_19036735, ZP_12085240, ZP_12040492, ZP_19048194, ZP_23086237, ZP_17844491, ZP_19053534, ZP_19059299, ZP_12012183, ZP_19064707, ZP_21366142, ZP_19070399, ZP_22557897, ZP_19081851, ZP_19075996, ZP_19109778, ZP_19357829, ZP_19092891, ZP_19087370, ZP_11992385, ZP_19104205, ZP_19098476, ZP_12025290, ZP_19115104, ZP_19363475, ZP_11973031, ZP_11981767, ZP_23055615, ZP_19120153, ZP_21371750, ZP_19131019, ZP_12000591, ZP_21250083, ZP_21255145, ZP_21260530, ZP_21265912, ZP_21271272, ZP_21276539, ZP_21350192, ZP_21281476, ZP_21344856, ZP_21287092, ZP_21292215, ZP_12312087, ZP_16210887, ZP_14332898, ZP_18669425, ZP_21297434, ZP_06662720, ZP_16877480, ZP_03062429, ZP_06657869, ZP_06653851, ZP_12078613, ZP_17680282, ZP_03030079, ZP_18647775, ZP_18972113, ZP_11952495, ZP_14466418, ZP_13797742, ZP_13803857, ZP_13809693, ZP_13815252, ZP_13820546, ZP_13826574, ZP_13831969, ZP_13837257, ZP_13842567, ZP_13848516, ZP_13853504, ZP_13858838, ZP_13864898, ZP_13870777, ZP_13876296, ZP_13881679, ZP_13886955, ZP_13891328, ZP_13896641, ZP_13901669, ZP_13906723, ZP_13911461, ZP_13916822, ZP_13922098, ZP_13927263, ZP_13932772, ZP_13937786, ZP_13943094, ZP_13948175, ZP_13953712, ZP_13544517, ZP_13549911, ZP_13555408, ZP_13561109, ZP_13565892, ZP_13571452, ZP_12404046, ZP_13576441, ZP_13581494, ZP_13586908, ZP_13592399, ZP_13598365, ZP_13604303, ZP_13609608, ZP_13615426, ZP_13621219, ZP_13626459, ZP_13632008, ZP_13637880, ZP_13643570, ZP_13649403, ZP_13654883, ZP_13660146, ZP_13665715, ZP_13670901, ZP_13676694, ZP_13681587, ZP_13687497, ZP_13693360, ZP_13698724, ZP_13703998, ZP_13709164, ZP_13714363, ZP_13719317, ZP_13724740, ZP_13730080, ZP_13735309, ZP_13740710, ZP_13746812, ZP_13752974, ZP_13758957, ZP_13764672, ZP_13769881, ZP_13775510, ZP_13780856, ZP_13786213, ZP_13791983, ZP_16883924, ZP_03052076, ZP_16814915, ZP_11501295, ZP_16820226, ZP_03047441, ZP_16825900, ZP_14817377, ZP_18700894, ZP_18706445, ZP_18712114, ZP_14811638, ZP_18015830, ZP_18717830, ZP_18723501, ZP_18729442, ZP_18734833, ZP_18740713, ZP_18746255, ZP_18751949, ZP_18021250, ZP_18757815, ZP_18763848, ZP_18769553, ZP_18775792, ZP_18781536, ZP_18787112, ZP_22728856, ZP_17981651, ZP_11679021, ZP_17953307, ZP_17947625, ZP_17987096, ZP_17970251, ZP_17976038, ZP_17998590, ZP_18004516, ZP_17992784, ZP_18010155, ZP_18653576, ZP_14885271, ZP_11495711, ZP_14890911, ZP_03034799, ZP_18572703, ZP_17814838, ZP_18561755, ZP_18566990, ZP_17820723, ZP_17832527, ZP_17838292, ZP_17826620, ZP_18584070, ZP_18578258, ZP_18602298, ZP_18798969, ZP_16259630, ZP_06990645, ZP_06649394, ZP_12268900, ZP_16827844, ZP_16803047, ZP_16809074, ZP_08384069, ZP_22684736, ZP_16886146, ZP_16839239, ZP_16969186, ZP_08378476, ZP_17679044, ZP_08343673, ZP_14463134, ZP_23045569, ZP_18976667, ZP_13509640, ZP_20400534, ZP_12019700, ZP_22078394, ZP_23014544, ZP_14432535, ZP_14434664, ZP_20043466, ZP_20202407, ZP_20207115, ZP_20211675, ZP_20216700, ZP_19613125, ZP_19896791, ZP_20221431, ZP_20226433, ZP_20049397, ZP_19901860, ZP_20231003, ZP_20054853, ZP_19906523, ZP_19615625, ZP_20235593, ZP_20240611, ZP_20060957, ZP_20245352, ZP_20250237, ZP_20254994, ZP_20259729, ZP_19620060, ZP_20264539, ZP_20269238, ZP_20065464, ZP_20068442, ZP_20273859, ZP_20278609, ZP_20282926, ZP_20073021, ZP_20077682, ZP_19910457, ZP_19915869, ZP_20081591, ZP_20287461, ZP_20086801, ZP_20089907, ZP_20292084, ZP_20296990, ZP_20301562, ZP_20098230, ZP_19921012, ZP_20306126, ZP_20100940, ZP_19624519, ZP_20310970, ZP_19927082, ZP_19929861, ZP_20315898, ZP_20106546, ZP_20320516, ZP_20324729, ZP_20329590, ZP_19934419, ZP_19630280, ZP_19939787, ZP_20123461, ZP_20334442, ZP_20128677, ZP_20338930, ZP_20343789, ZP_19664777, ZP_20348430, ZP_19958246, ZP_20353439, ZP_19668853, ZP_20136466, ZP_20134079, ZP_19674550, ZP_19678317, ZP_19683661, ZP_19962792, ZP_20118762, ZP_19688739, ZP_20109576, ZP_19692316, ZP_20113979, ZP_20142979, ZP_20147297, ZP_19699122, ZP_20151720, ZP_20156950, ZP_19703116, ZP_19708062, ZP_19713254, ZP_20160932, ZP_19718102, ZP_20167395, ZP_19723210, ZP_20170090, ZP_19727583, ZP_19731400, ZP_19737767, ZP_20174317, ZP_19742023, ZP_20179578, ZP_20184180, ZP_19635220, ZP_19746365, ZP_20188675, ZP_19751367, ZP_20193787, ZP_19755170, ZP_20196273, ZP_19989199, ZP_19761141, ZP_20358309, ZP_19768327, ZP_19771136, ZP_19774979, ZP_19780327, ZP_19785742, ZP_19976323, ZP_19640483, ZP_19644931, ZP_19649627, ZP_19984988, ZP_19597098, ZP_19654789, ZP_19972183, ZP_19980553, ZP_19659752, ZP_19967429, ZP_19994175, ZP_19790660, ZP_19999705, ZP_19795726, ZP_19601469, ZP_20005220, ZP_19800694, ZP_19805420, ZP_20009280, ZP_19810420, ZP_19817084, ZP_19820451, ZP_19824523, ZP_20013021, ZP_19606037, ZP_19829442, ZP_19834163, ZP_20017968, ZP_20021856, ZP_19838678, ZP_19843920, ZP_19844006, ZP_19945937, ZP_19847806, ZP_19852997, ZP_19858145, ZP_19863476, ZP_20027201, ZP_20033096, ZP_19867577, ZP_19873014, ZP_20362967, ZP_19877130, ZP_20036043, ZP_20367778, ZP_19882197, ZP_19887101, ZP_20372742, ZP_19948658, ZP_20377895, ZP_20039914, ZP_19891691, ZP_20382382, ZP_20387268, ZP_20391747, ZP_20396312, ZP_19954000, ZP_22360489, ZP_11502460, ZP_08348601, ZP_08354328, ZP_16847700, ZP_16865022, ZP_18636664, ZP_07098868, ZP_16440200, ZP_07134027, ZP_07165374, ZP_16434814, ZP_07102453, ZP_07211354, ZP_07690387, ZP_07245495, ZP_16447485, ZP_16452107, ZP_07168849, ZP_07140932, ZP_07197588, ZP_07144451, ZP_07192464, ZP_07117448, ZP_07175956, ZP_07150848, ZP_07180620, ZP_16461669, ZP_16455435, ZP_07182749, ZP_07220912, ZP_11541677, ZP_07122630, ZP_11534816, ZP_18695206, ZP_07448426, ZP_23180182, ZP_22419948, ZP_22713896, ZP_18596262, ZP_18793204, ZP_18590428, ZP_21019480, ZP_21895639, ZP_14386371, ZP_22568543, ZP_14392060, ZP_12479647, ZP_16994400, ZP_16990740, ZP_19303400, ZP_19313238, ZP_19316862, ZP_19322795, ZP_19328572, ZP_19330164, ZP_19338603, ZP_19343399, ZP_19354105, ZP_19348937, ZP_17000959, ZP_17010160, ZP_17015314, ZP_17021126, ZP_17028940, ZP_17034054, ZP_17035774, ZP_17042459, ZP_17049423, ZP_12512053, ZP_16978790, ZP_16985672, ZP_23973706, ZP_19379843, ZP_19390661, ZP_19394584, ZP_19401108, ZP_19406777, ZP_19384858, ZP_19409446, ZP_19254425, ZP_19373990, ZP_19259693, ZP_19417022, ZP_19420323, ZP_23115249, ZP_22122565, ZP_22675295, ZP_22475928, ZP_22298209, ZP_12452404, ZP_22180710, ZP_23157607, ZP_23983127, ZP_14614128, ZP_14399687, ZP_14403648, ZP_14616545, ZP_14412043, ZP_14416853, ZP_14603269, ZP_14606797, ZP_22787801, ZP_22491860, ZP_23978299, ZP_22269216, ZP_24043646, ZP_11878897, ZP_22193546, ZP_11657658, ZP_11666568, ZP_11661097, ZP_21968789, ZP_03084602, ZP_03260741, ZP_03257695, ZP_02776864, ZP_02802347, ZP_03252150, ZP_02783234, ZP_02796527, ZP_02827267, ZP_02815335, ZP_05941870, ZP_05948352, ZP_11873992, ZP_11898352, ZP_03445113, ZP_11883814, ZP_22986067, ZP_14623812, ZP_14429539, ZP_14632664, ZP_14637081, ZP_14422483, ZP_14643331, ZP_14333893, ZP_23075357, ZP_11888470, ZP_11897694, ZP_21015886, ZP_23069675, ZP_11507730, ZP_11513335, ZP_17867123, ZP_21302664, ZP_21312903, ZP_17873178, ZP_17879088, ZP_21307813, ZP_14772102, ZP_18613330, ZP_17884575, ZP_17890024, ZP_17895944, ZP_21318393, ZP_17686368, ZP_17901758, ZP_17907379, ZP_18555607, ZP_21355709, ZP_18685248, ZP_17913029, ZP_17849815, ZP_14778012, ZP_17918771, ZP_17924851, ZP_18625221, ZP_21323729, ZP_21328704, ZP_18619366, ZP_18607643, ZP_17855656, ZP_16266470, ZP_21334279, ZP_17861381, ZP_12095238, ZP_11523184, ZP_21900419, ZP_14228311, ZP_21893500, ZP_11927493, ZP_12253085, ZP_12232246, ZP_12237617, ZP_12259011, ZP_12263798, ZP_12274068, ZP_12279332, ZP_12316902, ZP_12285048, ZP_16843519, ZP_16975688, ZP_08364324, ZP_08369458, ZP_08374202, ZP_18630944, ZP_18674569, ZP_14782939, ZP_12074418, ZP_17930233, ZP_17935989, ZP_14800568, ZP_17941688, ZP_14806392, ZP_14790273, ZP_16833970, ZP_23108226, ZP_22279065, ZP_22162937, ZP_14794828, ZP_17964750, ZP_17959045, ZP_22010335, ZP_18664462, ZP_12289629, ZP_12915037, ZP_12680439, ZP_07593787, ZP_11673843, ZP_12615809, ZP_12586832, ZP_16854341, ZP_18241095, ZP_09806490, ZP_22103633, ZP_23226344, ZP_09457263, ZP_22909467, ZP_10139379, ZP_01437649, ZP_08328764, ZP_12702277, ZP_06832803, ZP_08899669, ZP_08316193, ZP_02165535, ZP_02168216, ZP_02165664, ZP_02167094, ZP_06548140, ZP_09344286, ZP_08303866, ZP_10888965, ZP_10892417, ZP_17091412, ZP_17091588, ZP_17096794, ZP_17102750, ZP_17108668, ZP_17112244, ZP_17112416, ZP_16166000, ZP_16167938, ZP_23926727, ZP_23932546, ZP_23921938, ZP_18256069, ZP_21738335, ZP_23428850, ZP_14534145, ZP_14539767, ZP_14544307, ZP_14550017, ZP_14556598, ZP_14562207, ZP_14567077, ZP_14572300, ZP_14489374, ZP_14578801, ZP_14586232, ZP_14588889, ZP_14596727, ZP_14494636, ZP_14500390, ZP_14505545, ZP_14512625, ZP_14517315, ZP_14523855, ZP_14529212, ZP_18351091, ZP_06016571, ZP_23757869, ZP_16339920, ZP_16348599, ZP_18996111, ZP_23555547, ZP_18479119, ZP_18485339, ZP_18489742, ZP_18495370, ZP_01550467, ZP_06187831, ZP_03698916, ZP_23009604, ZP_01012866, ZP_09292499, ZP_09091185, ZP_09091462, ZP_09091977, ZP_23965869, ZP_10016760, ZP_10236449, ZP_11154204, ZP_11156556, ZP_11123981, ZP_11124054, ZP_11124593, ZP_02151616, ZP_02151802, ZP_02153141, ZP_02153710, ZP_00952894, ZP_00999364, ZP_01155530, ZP_09515611, ZP_09517577, ZP_11144940, ZP_10965015, ZP_10967316, ZP_10967741, ZP_23284313, ZP_23286121, ZP_04679364, ZP_04682785, ZP_21144788, ZP_21145713, ZP_08274651, ZP_08665683, ZP_03825849, ZP_03829067, ZP_03831355, ZP_15541619, ZP_01444033, ZP_01444475, ZP_10585487, ZP_10585767, ZP_10585796, ZP_10292367, ZP_09053510, ZP_09055319, ZP_11910195, ZP_11913618, ZP_11915053, ZP_11917277, ZP_11919899, ZP_11920807, ZP_21931284, ZP_21934618, ZP_21935368, ZP_04932828, ZP_04933614, ZP_07792362, ZP_07796491, ZP_15611574, ZP_15613913, ZP_15614846, ZP_15617345, ZP_15620047, ZP_15621043, ZP_15626233, ZP_15626532, ZP_15627399, ZP_04927593, ZP_04928314, ZP_15630423, ZP_15632671, ZP_15633481, ZP_14645325, ZP_14645576, ZP_14648596, ZP_15636357, ZP_15638521, ZP_15639363, ZP_13147464, ZP_13148602, ZP_13152507, ZP_13154253, ZP_13154991, ZP_13158096, ZP_18356107, ZP_18356897, ZP_22977693, ZP_22986483, ZP_22989475, ZP_06876868, ZP_06879176, ZP_06879970, ZP_01364507, ZP_01365250, ZP_14278695, ZP_14281589, ZP_15962450, ZP_15963229, ZP_21956021, ZP_10474525, ZP_10477865, ZP_16385647, ZP_16387610, ZP_18876575, ZP_10434982, ZP_10436717, ZP_15602014, ZP_15603480, ZP_20919135, ZP_20919209, ZP_17085230, ZP_17669729, ZP_17671250, ZP_17673916, ZP_18348381, ZP_17665212, ZP_17668197, ZP_07774203, ZP_07777648, ZP_10848294, ZP_10991074, ZP_10991684, ZP_10992684, ZP_11208847, ZP_10595198, ZP_10703673, ZP_10698961, ZP_10684396, ZP_10688685, ZP_10680436, ZP_10678131, ZP_10678316, ZP_10666570, ZP_10668140, ZP_10665043, ZP_10665241, ZP_10659014, ZP_10660167, ZP_10651483, ZP_10647013, ZP_10647511, ZP_10638917, ZP_10633164, ZP_10627946, ZP_10629197, ZP_10624528, ZP_10613930, ZP_10616657, ZP_10610100, ZP_10603087, ZP_11260304, ZP_11262112, ZP_10148993, ZP_10151159, ZP_11115054, ZP_10430676, ZP_10431379, ZP_21058861, ZP_09286956, ZP_23916153, ZP_15975801, ZP_11185327, ZP_11189205, ZP_21946221, ZP_10144823, ZP_10142278, ZP_07261743, ZP_16731224, ZP_16734891, ZP_16714321, ZP_16714957, ZP_16656647, ZP_16658490, ZP_16660441, ZP_06477994, ZP_06480927, ZP_06458590, ZP_06459384, ZP_06461229, ZP_16729469, ZP_17804350, ZP_17806833, ZP_17809007, ZP_17811230, ZP_21128467, ZP_20901923, ZP_20904852, ZP_20921770, ZP_20925904, ZP_16703899, ZP_16705491, ZP_11564662, ZP_11565523, ZP_11568926, ZP_11569639, ZP_16481020, ZP_16482301, ZP_16685979, ZP_16670194, ZP_16671475, ZP_16720724, ZP_16722238, ZP_16722992, ZP_23736261, ZP_16707695, ZP_16710898, ZP_16677062, ZP_16678221, ZP_16664008, ZP_16666439, ZP_04586115, ZP_04586362, ZP_04592751, ZP_16695770, ZP_16696482, ZP_16697275, ZP_16740370, ZP_16742194, ZP_05636126, ZP_05638507, ZP_07250213, ZP_07250594, ZP_07253378, ZP_07231628, ZP_07234311, ZP_07234854, ZP_07256847, ZP_07257182, ZP_07259719, ZP_03394848, ZP_03395623, ZP_03398368, ZP_08137751, ZP_08143015, ZP_11286740, ZP_11288242, ZP_05085253, ZP_05086399, ZP_10987261, ZP_07674129, ZP_16326608, ZP_10536310, ZP_10536361, ZP_10538601, ZP_10539281, ZP_10835810, ZP_10836503, ZP_10836910, ZP_10837140, ZP_10839016, ZP_10839301, ZP_10839334, ZP_14743692, ZP_14744101, ZP_14745545, ZP_14748191, ZP_10733727, ZP_10733957, ZP_10735329, ZP_10736775, ZP_10737943, ZP_11031148, ZP_11031585, ZP_11034186, ZP_11034307, ZP_11035907, ZP_11036389, ZP_11037253, ZP_03511148, ZP_03511536, ZP_03512608, ZP_03512817, ZP_03504117, ZP_03504832, ZP_03506871, ZP_03525586, ZP_03527054, ZP_03528156, ZP_03528942, ZP_03530449, ZP_11959391, ZP_11961595, ZP_11962382, ZP_11962569, ZP_11962723, ZP_11964051, ZP_03521861, ZP_03522316, ZP_03523203, ZP_03515878, ZP_03515954, ZP_03517556, ZP_03517836, ZP_03518826, ZP_03519465, ZP_03519740, ZP_03500105, ZP_03501580, ZP_03503438, ZP_03503484, ZP_18309091, ZP_18310498, ZP_18312041, ZP_18312258, ZP_18313091, ZP_18313312, ZP_18316119, ZP_18316814, ZP_18319240, ZP_18319861, ZP_18321094, ZP_18321842, ZP_18322091, ZP_18338365, ZP_18338672, ZP_18338905, ZP_18339680, ZP_18342018, ZP_18343786, ZP_18300908, ZP_18302246, ZP_18303648, ZP_18303870, ZP_18306335, ZP_18306456, ZP_18332973, ZP_18334384, ZP_18334838, ZP_18336174, ZP_18295445, ZP_18296542, ZP_18297352, ZP_18297591, ZP_18299464, ZP_18300740, ZP_11198150, ZP_11199545, ZP_11199680, ZP_11266574, ZP_11263452, ZP_11265017, ZP_11265389, ZP_11267036, ZP_11268438, ZP_11268840, ZP_13489635, ZP_13489748, ZP_13492373, ZP_13492641, ZP_13493166, ZP_16033168, ZP_16033190, ZP_16034840, ZP_16035325, ZP_16035984, ZP_16036726, ZP_16038092, ZP_05122922, ZP_05074601, ZP_01742323, ZP_05077707, ZP_08414488, ZP_08414761, ZP_05843094, ZP_05843466, ZP_07660200, ZP_07661454, ZP_01903392, ZP_01752233, ZP_05099219, ZP_01057460, ZP_01753468, ZP_01755555, ZP_01756179, ZP_01034229, ZP_00961191, ZP_00960743, ZP_01879559, ZP_08402070, ZP_05089864, ZP_08860748, ZP_01744727, ZP_01744778, ZP_01748294, ZP_10803205, ZP_17128166, ZP_23063520, ZP_02574613, ZP_12111562, ZP_20947274, ZP_20953637, ZP_20943910, ZP_12122250, ZP_22511556, ZP_09760024, ZP_21389577, ZP_09763785, ZP_21388636, ZP_23943280, ZP_22704799, ZP_22108465, ZP_22920594, ZP_21419922, ZP_20741019, ZP_21416171, ZP_21400060, ZP_22791110, ZP_22643880, ZP_22581881, ZP_15874808, ZP_20515156, ZP_20841012, ZP_21409831, ZP_15832726, ZP_15896081, ZP_20853726, ZP_20869095, ZP_20752301, ZP_20756235, ZP_20825807, ZP_20762790, ZP_20819646, ZP_20664436, ZP_15897173, ZP_15837591, ZP_15841661, ZP_20850638, ZP_20681974, ZP_15828160, ZP_20674308, ZP_20678891, ZP_20686702, ZP_20864189, ZP_15808876, ZP_20691660, ZP_15845040, ZP_15851241, ZP_20665947, ZP_20735517, ZP_15812801, ZP_15852887, ZP_15860181, ZP_15817942, ZP_20767581, ZP_20819409, ZP_20769814, ZP_20775677, ZP_20782037, ZP_20783537, ZP_20790056, ZP_20831656, ZP_20792458, ZP_15890864, ZP_20797768, ZP_20802933, ZP_20809256, ZP_15886071, ZP_20812255, ZP_20834726, ZP_15824002, ZP_15862419, ZP_15866377, ZP_15871320, ZP_20860334, ZP_15881617, ZP_20583528, ZP_20588784, ZP_20631200, ZP_20600839, ZP_20575315, ZP_20569246, ZP_20557364, ZP_20606143, ZP_20609909, ZP_20635934, ZP_20590974, ZP_20551337, ZP_20564049, ZP_20615106, ZP_20617457, ZP_20542030, ZP_20641915, ZP_20546715, ZP_20537304, ZP_20644901, ZP_20577411, ZP_20650359, ZP_20595669, ZP_20654108, ZP_20660701, ZP_20624853, ZP_20561849, ZP_20627501, ZP_20530346, ZP_20533388, ZP_20521706, ZP_20746097, ZP_20703864, ZP_20730279, ZP_20709085, ZP_20718180, ZP_20700747, ZP_21423943, ZP_20845357, ZP_21405749, ZP_20694484, ZP_21394618, ZP_20722434, ZP_20728939, ZP_21383826, ZP_09768319, ZP_12127347, ZP_12132565, ZP_21991542, ZP_02681739, ZP_23095639, ZP_14258984, ZP_14274014, ZP_14269032, ZP_14268120, ZP_14257339, ZP_16014696, ZP_16020280, ZP_16025294, ZP_16030359, ZP_02669729, ZP_12141222, ZP_09725255, ZP_12142914, ZP_03221863, ZP_12148644, ZP_03074890, ZP_12153773, ZP_22482491, ZP_12158610, ZP_11751713, ZP_11835007, ZP_11839209, ZP_11755223, ZP_11843111, ZP_11689703, ZP_11784944, ZP_11786072, ZP_11777409, ZP_11762129, ZP_11766171, ZP_11791166, ZP_11697204, ZP_11698403, ZP_11706714, ZP_11811281, ZP_11708934, ZP_11711554, ZP_11718542, ZP_11800187, ZP_11814153, ZP_11795210, ZP_11803741, ZP_13053038, ZP_11771208, ZP_11737746, ZP_11730140, ZP_13059103, ZP_13060247, ZP_13063967, ZP_13068657, ZP_13075852, ZP_11847130, ZP_11852237, ZP_11859566, ZP_11862042, ZP_11866802, ZP_13093733, ZP_11871192, ZP_11743579, ZP_11817546, ZP_11824375, ZP_11829460, ZP_11830672, ZP_11774774, ZP_11721547, ZP_11726555, ZP_12163786, ZP_11747466, ZP_11733438, ZP_13399447, ZP_13399738, ZP_13345294, ZP_13350482, ZP_13413614, ZP_13418063, ZP_13352596, ZP_13408546, ZP_13370426, ZP_13328394, ZP_13365050, ZP_13395428, ZP_13339033, ZP_13378116, ZP_13381604, ZP_13369172, ZP_13331538, ZP_13316900, ZP_13334069, ZP_13320949, ZP_13356396, ZP_13405347, ZP_13422628, ZP_13391512, ZP_13388549, ZP_23303102, ZP_23299090, ZP_14319951, ZP_14315096, ZP_23312617, ZP_23307647, ZP_02696443, ZP_13079502, ZP_20873048, ZP_12169582, ZP_03163551, ZP_02346573, ZP_02664244, ZP_12175104, ZP_16315386, ZP_04656314, ZP_22968329, ZP_22754922, ZP_03346391, ZP_03359106, ZP_03371957, ZP_06543670, ZP_03380389, ZP_22862619, ZP_18951889, ZP_18956801, ZP_18961602, ZP_16372554, ZP_16377353, ZP_18937364, ZP_18942229, ZP_18966780, ZP_18947124, ZP_18927860, ZP_18932575, ZP_09722303, ZP_22240527, ZP_22690353, ZP_22542146, ZP_12180241, ZP_12185392, ZP_03217677, ZP_12191166, ZP_02834990, ZP_23050340, ZP_23051045, ZP_23058509, ZP_06189549, ZP_06192411, ZP_16217887, ZP_16219274, ZP_10109684, ZP_10112736, ZP_12330836, ZP_14853112, ZP_12339207, ZP_14847621, ZP_11649018, ZP_08391326, ZP_03067206, ZP_12321375, ZP_14878923, ZP_11641213, ZP_14873099, ZP_16122645, ZP_12382383, ZP_14822574, ZP_12392200, ZP_11530493, ZP_12387425, ZP_18262935, ZP_12880491, ZP_14827568, ZP_11652589, ZP_12474792, ZP_14833250, ZP_12351607, ZP_12366469, ZP_12361818, ZP_12372219, ZP_14838002, ZP_14843625, ZP_12377423, ZP_12356817, ZP_14858995, ZP_11432682, ZP_14863777, ZP_11527603, ZP_12884760, ZP_05787659, ZP_05739123, ZP_05743091, ZP_05742929, ZP_12973745, ZP_12975160, ZP_12977750, ZP_10218177, ZP_10218231, ZP_18901369, ZP_18901429, ZP_10873066, ZP_10874324, ZP_10421857, ZP_10958324, ZP_08390518, ZP_01304219, ZP_18105262, ZP_23765349, ZP_23295302, ZP_05133878, ZP_00956046, ZP_00953750, ZP_00963648, ZP_00948295, ZP_05341753, ZP_10569396, ZP_04417599, ZP_20892110, ZP_01975034, ZP_04399658, ZP_16954309, ZP_04415958, ZP_04395477, ZP_05420312, ZP_18028022, ZP_15767404, ZP_14349661, ZP_15770659, ZP_18032633, ZP_15774669, ZP_15777632, ZP_18036420, ZP_15782681, ZP_18043077, ZP_15786304, ZP_18043766, ZP_06050237, ZP_22126113, ZP_12941419, ZP_17706249, ZP_17745167, ZP_17134332, ZP_15790245, ZP_17135211, ZP_17138850, ZP_12946654, ZP_12948808, ZP_17145258, ZP_17148919, ZP_17747330, ZP_16960715, ZP_18047483, ZP_16932275, ZP_18054097, ZP_18057836, ZP_12954168, ZP_15796291, ZP_18058693, ZP_16936003, ZP_17149965, ZP_12458498, ZP_17710749, ZP_18080549, ZP_18088432, ZP_12956270, ZP_17728386, ZP_17761938, ZP_21072519, ZP_21076235, ZP_21079937, ZP_21083647, ZP_17768588, ZP_16939658, ZP_21087188, ZP_21088041, ZP_17729299, ZP_21095805, ZP_21099426, ZP_21103015, ZP_18091985, ZP_12463491, ZP_18096237, ZP_16949559, ZP_06029397, ZP_07009474, ZP_05239402, ZP_01978060, ZP_01957060, ZP_01972379, ZP_23819767, ZP_22115591, ZP_22889871, ZP_23826872, ZP_23830915, ZP_23834781, ZP_23837048, ZP_23841731, ZP_23844510, ZP_23849081, ZP_23853351, ZP_23855300, ZP_23859258, ZP_23864542, ZP_23866432, ZP_23870595, ZP_23876601, ZP_23879441, ZP_21736076, ZP_23898247, ZP_23901319, ZP_23883364, ZP_23885228, ZP_23892650, ZP_23905397, ZP_19369006, ZP_06036848, ZP_06941982, ZP_04407138, ZP_22171406, ZP_01679712, ZP_22341440, ZP_22255473, ZP_05886742, ZP_05882859, ZP_13082871, ZP_13085550, ZP_10263284, ZP_06489148, ZP_06486081, ZP_09882977, ZP_06729588, ZP_06703918, ZP_08181953, ZP_08187599, ZP_08179515, ZP_04626984, ZP_04627356, ZP_04630091, ZP_14762248, ZP_04633048, ZP_04635535, ZP_04637801, ZP_04639446, ZP_04639597, ZP_02307757, ZP_02307842, ZP_02222949, ZP_15053299, ZP_15086991, ZP_15321573, ZP_15092080, ZP_15106511, ZP_15120547, ZP_15168717, ZP_15178388, ZP_15254603, ZP_04611213, ZP_04615773, ZP_09390038, YP_006819568, YP_006819571, YP_001834028, YP_001834031, YP_776118, YP_776119, YP_001811424, YP_001811425, YP_006617700, YP_006617701, YP_006336045, YP_006336046, YP_105810, YP_105811, YP_001023905, YP_001023907, YP_001583713, YP_001583714, YP_001949162, YP_001949163, YP_006277403, YP_006277404, YP_001074949, YP_001074952, YP_337395, YP_337392, YP_001061999, YP_001062001, YP_006657804, YP_006657807, YP_110689, YP_110690, YP_439945, YP_439944, YP_001117758, YP_001117759, YP_001533158, YP_001533162, YP_002967090, YP_002967091, YP_004864636, YP_004864637, YP_004118441, YP_004118442, YP_004302368, YP_471756, YP_471757, YP_002824988, YP_002824989, YP_353196, YP_353197, YP_001167906, YP_001167907, YP_001043639, YP_001043640, YP_002525799, YP_002525800, YP_005187831, YP_005187832, YP_006575836, YP_006575837, YP_006395891, YP_006395892, YP_001326132, YP_001326133, NP_384934, NP_384935, YP_004547911, YP_004547912, YP_005712626, YP_005712627, YP_005718014, YP_005718017, YP_007189392, YP_007189393, YP_006814421, YP_006814427, YP_006839353, YP_006839354, YP_005719023, YP_005719024, YP_004556252, YP_004556253, YP_003543918, YP_003543919, YP_004831217, YP_004831218, YP_008327691, YP_008327692, YP_007921919, YP_007921920, YP_007574091, YP_007574092, YP_007592445, YP_007592446, ZP_11558703, ZP_08946313, ZP_05825506, ZP_12982948, ZP_12982950, ZP_10083817, ZP_10085126, ZP_02890473, ZP_02890474, ZP_02909023, ZP_02909024, ZP_21163032, ZP_21163033, ZP_16302379, ZP_16302380, ZP_21172999, ZP_21173000, ZP_04947670, ZP_04947671, ZP_04972827, ZP_04972829, ZP_04885800, ZP_04885857, ZP_04906988, ZP_04906990, ZP_00438872, ZP_00438873, ZP_04911002, ZP_04911004, ZP_02265153, ZP_04819839, ZP_15916581, ZP_15916591, ZP_03582566, ZP_03582567, ZP_03569116, ZP_03573592, ZP_03573593, ZP_03575762, ZP_03579244, ZP_03579245, ZP_15928425, ZP_15928426, ZP_02365338, ZP_02365340, ZP_02358275, ZP_02358277, ZP_13101320, ZP_13101321, ZP_13101544, ZP_04810534, ZP_04811008, ZP_13108573, ZP_13108574, ZP_13108795, ZP_13114757, ZP_13114758, ZP_13114982, ZP_04889631, ZP_04889633, ZP_04953252, ZP_04954547, ZP_12969619, ZP_12969620, ZP_12969852, ZP_13120152, ZP_13120153, ZP_13120350, ZP_04967418, ZP_04967421, ZP_04967686, ZP_03450344, ZP_03450792, ZP_23812010, ZP_23812012, ZP_04521180, ZP_04522237, ZP_04523221, ZP_03790460, ZP_03790628, ZP_04896207, ZP_04896210, ZP_04899185, ZP_04899688, ZP_05591373, ZP_05591374, ZP_18328849, ZP_18328850, ZP_11930953, ZP_11930954, ZP_02380647, ZP_02380648, ZP_18917788, ZP_18917789, ZP_21175421, ZP_08017459, ZP_08017460, ZP_12696683, ZP_12696684, ZP_10236911, ZP_10236914, ZP_11959263, ZP_18300816, ZP_01740253, ZP_01740254, ZP_08412871, ZP_08412872, ZP_12977045, ZP_12977046, ZP_10218436, ZP_10218437, ZP_18902225, ZP_18902228, ZP_01303636, ZP_01303637 |
