# Supplementary material for: Census of solo LuxR genes in prokaryotic genomes
Source: Front Cell Infect Microbiol. 2015 Mar 12;5:20. doi: 10.3389/fcimb.2015.00020 (PMC4357305; doi:10.3389/fcimb.2015.00020)
Supplement: Supplementary file 2 [file Table2.DOCX]

Table 2.Accession IDs of genes constituting tandem and divergent twin *luxR* –*luxR* topologies.

| **Tandem *luxR-luxR* topologies** |
| --- |
| NP_384934, NP_384935, YP_471756, YP_471757, YP_001326132, YP_001326133, YP_002824988, YP_002824989, YP_002967090, YP_002967091, YP_004118441, YP_004118442, ZP_08017459, ZP_08017460, YP_004547911, YP_004547912, YP_004864636, YP_004864637, YP_005187831, YP_005187832, YP_005712626, YP_005712627, YP_005719023, YP_005719024, YP_006395891, YP_006395892, YP_006575836, YP_006575837, YP_006839353, YP_006839354, ZP_12696683, ZP_12696684, ZP_12977045, ZP_12977046, ZP_18917788, ZP_18917789, YP_007189392, YP_007189393, YP_007574091, YP_007574092 |
| **Divergent*luxR-luxR* topologies** |
| YP_105810, YP_105811, YP_110689, YP_110690, ZP_00438872, YP_337395, YP_337392, YP_439945, YP_439944, YP_776118, YP_776119, YP_001023905, YP_001023907, YP_001061999, YP_001062001, YP_001074949, YP_001074952, YP_001117758, YP_001117759, YP_001583713, YP_001583714, ZP_02265153, ZP_02358275, ZP_02358277, ZP_02365338, ZP_02365340, ZP_02380647, ZP_02380648, ZP_02890473, ZP_02890474, ZP_02909023, ZP_02909024, YP_001811424, YP_001811425, YP_001949162, YP_001949163, ZP_03450344, ZP_03450792, ZP_03573592, ZP_03573593, ZP_03579244, ZP_03579245, ZP_03582566, ZP_03582567, ZP_04521180, ZP_04522237, ZP_00438873, ZP_04819839, ZP_04885800, ZP_04885857, ZP_04889631, ZP_04889633, ZP_04899185, ZP_04899688, ZP_04906988, ZP_04906990, ZP_04911002, ZP_04911004, ZP_04947670, ZP_04947671, ZP_04972827, ZP_04972829, ZP_05591373, ZP_05591374, YP_006277403, YP_006277404, YP_006336045, YP_006336046, YP_006617700, YP_006617701, YP_006657804, YP_006657807, ZP_11930953, ZP_11930954, ZP_12969619, ZP_12969620, ZP_13101320, ZP_13101321, ZP_13108573, ZP_13108574, ZP_13114757, ZP_13114758, ZP_13120152, ZP_13120153, ZP_15916581, ZP_15916591, ZP_15928425, ZP_15928426, ZP_16302379, ZP_16302380, ZP_18328849, ZP_18328850, ZP_21163032, ZP_21163033, ZP_21172999, ZP_21173000, ZP_23812010, ZP_23812012, YP_007921919, YP_007921920, YP_008327691, YP_008327692 |
