# Supplementary material for: Census of solo LuxR genes in prokaryotic genomes
Source: Front Cell Infect Microbiol. 2015 Mar 12;5:20. doi: 10.3389/fcimb.2015.00020 (PMC4357305; doi:10.3389/fcimb.2015.00020)
Supplement: Supplementary file 5 [file DataSheet3.DOCX]

1. **Searching for disulfide bridge candidates**

In order to detect disulfide bridge candidate residues, we aligned the sequences of each clade (Figure 3 of the paper) to three PDB templates which corresponded to LuxR proteins. The idea was to match conserved Cysteine positions of LuxR proteins to the templates, and obtain the distances among them, to see if they come close enough (<=7Å) to each other to make it possible for disulfide bridges to form. Once we know the corresponding residues on the templates, we calculated the distances between them using PdbEditor (http://pdbeditor.sourceforge.net/).

We also checked distances between all possible combinations of residues residing within 5 residues distance from each conserved cysteine positions, to make the search more exhaustive.

Only the conserved cysteins of LuxRs from *Pseudomallei* solo clade (Figure 1) were found to satisfy these conditions.

3D images (Figure 4 of publication) of templates with the positions satisfying above described conditions marked on them were generated using PyMol (http://www.pymol.org/)
