# Supplementary figures and images for: Census of solo LuxR genes in prokaryotic genomes
Source: Front Cell Infect Microbiol. 2015 Mar 12;5:20. doi: 10.3389/fcimb.2015.00020 (PMC4357305; doi:10.3389/fcimb.2015.00020)

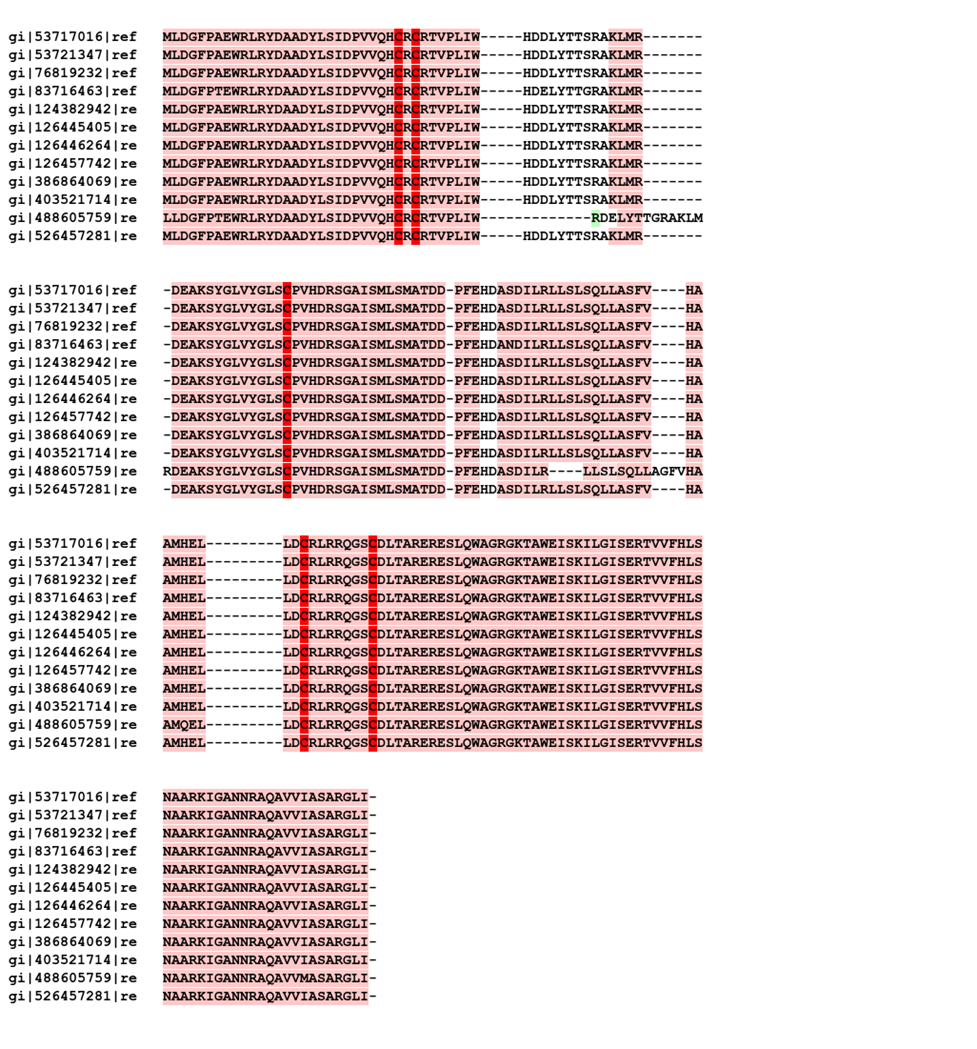

Supplement: Supplementary file 6 [file Image1.TIF]
